# Three Dimensional Assessment of the Pharyngeal Airway in Individuals with Non-Syndromic Cleft Lip and Palate

**DOI:** 10.1371/journal.pone.0043405

**Published:** 2012-08-29

**Authors:** Tracy Cheung, Snehlata Oberoi

**Affiliations:** 1 University of California Los Angeles, Los Angeles, California, United States of America; 2 Division of Orthodontics, Department of Orofacial Sciences, School of Dentistry, University of California San Francisco, San Francisco, California, United States of America; Hospital of the University of Pennsylvania, United States of America

## Abstract

**Introduction:**

Children with cleft lip and palate (CLP) are known to have airway problems. Previous studies have shown that individuals with CLP have a 30% reduction in nasal airway size compared to non-cleft controls. No reports have been found on cross-sectional area and volume of the pharyngeal airway in clefts. Introduction of Cone-Beam CT (CBCT) and imaging software has facilitated generation of 3D images for assessment of the cross-sectional area and volume of the airway.

**Objective:**

To assess the pharyngeal airway in individuals with CLP using CBCT by measuring volume and smallest cross-sectional areas and compare with 19 age- and sex-matched non-cleft controls.

**Methods:**

Retrospective study of CBCT data of pre-adolescent individuals (N = 19, Mean age = 10.6, 7 females, 12 males, UCLP = 6, BCLP = 3) from the Center for Craniofacial Anomalies. Volumetric analysis was performed using image segmentation features in CB Works 3.0. Volume and smallest cross-sectional were studied in both groups. Seven measurements were repeated to verify reliability using Pearson correlation coefficient. Volume and cross-sectional area differences were analyzed using paired t-tests.

**Results:**

The method was found to be reliable. Individuals with CLP did not exhibit smaller total airway volume and cross sectional area than non-CLP controls.

**Conclusion:**

3D imaging using CBCT and CB Works is reliable for assessing airway volume. Previous studies have shown that the nasal airway is restricted in individuals with CLP. In our study, we found that the pharyngeal airway is not compromised in these individuals.

## Introduction

Birth defects affect approximately 3% of all births and contribute substantially to childhood morbidity in the United States [1]. Orofacial defects like cleft lip and/or palate (CLP) occur in an estimated 6,800 children annually, making it the most common birth defect in the United States [2]. Anatomical abnormalities associated with CLP increase the risk of airway complications [3].

CLP are frequently associated with nasal abnormalities such as septal deviation, nostril atresia, turbinate hypertrophy, maxillary constriction, vomerine spurs, and alar constriction [4], [5], [6], [7], [8]. These abnormalities are attributed in part to the congenital defect itself and partly to the surgeries done to repair the orofacial defect [9], [10]. Collectively, the nasal abnormalities tend to reduce the dimensions of the nasal cavity and lower airway function [6], [11].

Airway patency has been evaluated by two-dimensional (2D) radiographic imaging such as lateral and anterior-posterior cephalometric films and functional studies such as rhinomanometry and plethysmography [12], [13], [14], [15]. A rhinomanometry study showed that individuals with bilateral CLP had a 41% reduction in nasal airway compared to a 19% reduction in individuals with unilateral CLP [15]. Plethysmography showed that individuals with CLP had a 30% reduction in the nasal airway compared to non-cleft controls [12]. Lateral cephalometric films of children with CLP were compared with non-cleft controls and showed a reduction in the nasopharyngeal bony framework and pharyngeal airway [16]. Furthermore, a reduction in the upper airway in juveniles with CLP was found when compared to gender and age matched non-cleft controls and were found to persist through adolescence when lateral cephalometric films were compared [13].

Until recently, diagnostic imaging of the airway has been limited to 2D studies. Two-dimensional imaging using lateral head films is useful for analyzing airway size in the sagittal plane, but does not depict the three-dimensional anatomy. The physiologically most relevant information is obtained from axial images which are perpendicular to the direction of airflow, but the axial plane is not visualized on lateral cephalograms [17]. In addition, lateral cephalometric films have many limitations, such as image enlargement and distortion, structure overlap, limited identifiable landmarks, and positioning problems that may adversely affect image quality [18]. In contract, three-dimensional techniques like Computed Tomography (CT) or Cone Beam Computed Tomography (CBCT) data provide an accurate 3D assessment of the airway in all three planes; coronal, sagittal and axial [17]. However, the radiation dose for standard CT scan of the maxillofacial region is high and repeated scanning is a concern. Medium field-of-view CBCT radiation ranged from 69 to 560 microsieverts (mSv); whereas, a similar field-of-view medical CT produced 860 mSv [19].

CBCT has been shown to be effective in localization of impacted teeth and in assessing the outcome of alveolar bone grafting and the eruption path of the canine in grafted alveolar clefts [20], [21], [22]. Previous studies have shown that 3D imaging using CBCT is a simple and effective method to accurately analyze the airway [23], [24]. Advantages of CBCT include X-ray beam limitation, image accuracy, rapid scan time, dose reduction, display modes unique to maxillofacial imaging, and reduced image artifact [25]. Recently, there have been multiple studies using CBCT to assess the airway in relation to facial morphology in individuals with sleep apnea [26], [27]. To date, we have not found any published CBCT studies of the airway of individuals with CLP, despite the frequently occurring airway problems in these individuals [4], [5], [6], [7]. We hypothesized that individuals with CLP have smaller pharyngeal cross-sectional areas and volumes compared to non-cleft controls.

## Methods

This was retrospective study of subjects recruited from the Craniofacial Center and Orthodontic clinic. Written informed consent was obtained for all participants of the study which was approved by CHR. We obtained ethics approval for our study from the ethics committee at UCSF, (CHR # h44601-34031-02). Previous published data on volume in non cleft individuals report an average volume of 20.9 mm^3^ with a S.D. of 3.6 mm^3^. We made the assumption that the airway would be 10% smaller in CL/P with the same S.D. as the control. This calculation resulted in a sample size of 29, in order to detect a difference.

The experimental group included 19 individuals (12 male and 7 female with CLP; 16 with unilateral and 3 with bilateral complete clefts). The exclusion criteria included clefting associated with diagnosed syndromes and no prior adenoidectomy and/or tonsillectomy. All individuals with CLP had maxillary expansion and alveolar bone grafting. The control group included 19 individuals who were gender, age, and Sella-Nasion (SN) matched with the experimental group. The inclusion criteria included Angle Class I malocclusion with beginning CBCT records for starting orthodontic treatment and no prior orthodontic treatment, no habitual mouth breathing recorded and no prior adenoidectomy and/or tonsillectomy.

CBCT scans with 0.4 mm slices were obtained at one time point on the Hitachi MercuRay CBCT machine (Hitachi Medical Corporation, Japan). Scans were standardized with an 8×8-inch field of view and contained 512 axial sections in 0.4 mm thick increments. DICOM files were imported into CB Works 3.0 (Seoul, Korea), software capable of volume rendering. First, a CBCT technician de-identified the files, removing name, sex and date of birth from the DICOM. Next, files were reoriented to a standardized view. In the sagittal view, the vertebral column was realigned perpendicular to the horizontal axis. If the vertebral column was curved, the pharynx was reoriented perpendicular to the horizontal axis. It was preferred to use the vertebral column as a landmark because it is a hard tissue.

**Figure 1:**
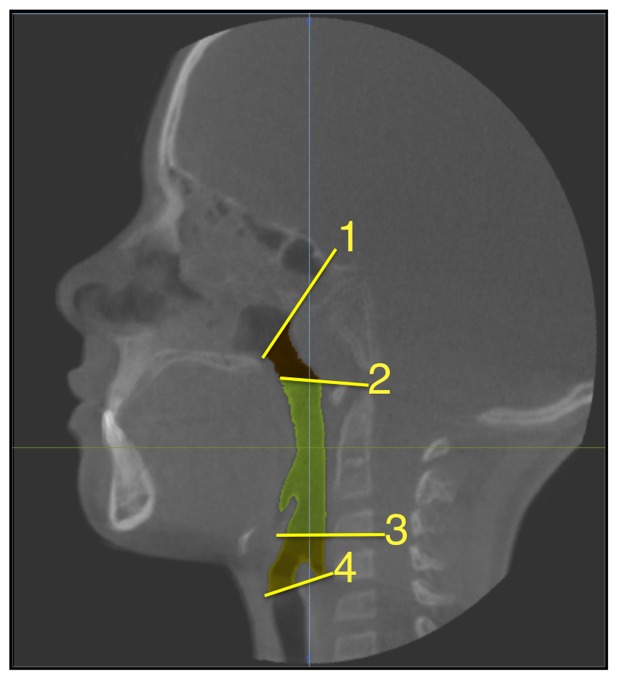
Landmarks of pharyngeal airway as shown in sagittal view. 1) Center of Sella to Posterior Nasal Spine 2) Hard Palate 3) Vallecula 4) Junction of larynx and esophagus.

The airway was segmented and volume rendered in multiplanar reconstruction (MPR) mode. The threshold tool was used to select the airway space. The histogram was set to include data within the boundaries of 1025 to −524 HU. These values were saved as a preset in CB Works. Next, the Volume of Interest (VOI) tool was used to grossly remove data outside of the airway. The airway data were further refined and superior and inferior borders were defined using the Regional Edit tool. The superior border was defined as a line connecting Sella and Posterior Nasal Spine. The inferior border was defined as a line connecting the most anterior region of the airway formed by the thyroid cartilage to the transverse arytenoid muscle at the level where the esophagus splits from the airway (Fig. 1). The SSD function was used to create a 3D rendering of the airway space. The volume (cm^3^) and smallest cross sectional area was determined from the total airway segmentation.

In order to define the location of the smallest cross sectional area, the airway was further segmented into three sections: nasopharynx, oropharynx and hypopharynx. The superior border of the nasopharynx was defined as the posterior choana and inferiorly as the horizontal line along the hard and soft palates. The oropharynx was defined superiorly by the soft palate and inferiorly by the vallecula. The hypopharynx was defined superiorly by the hyoid bone and vallecula and inferiorly by the junction of the larynx and esophagus (Fig. 1).

The linear measurement tool in the CB Works 3.0 software was used to determine SN in order to properly match the experimental and control groups.

## Results

The Pearson Correlation Coefficient and Lin Concordance were used to test the reliability of the methods used in choosing landmarks that further lead to measurements of airway volumes and cross sectional areas. Seven randomly selected cases were re-studied and airway volumes and cross sectional areas re-measured with a Pearson Correlation Coefficient of .99 and a Lin Concordance of .99 for both measurements.

The mean anterior cranial base length (SN) was 55 and 56 mm, respectively, for the cleft and non-cleft individuals, indicating no significant size different between the groups.

**Figure 2:**
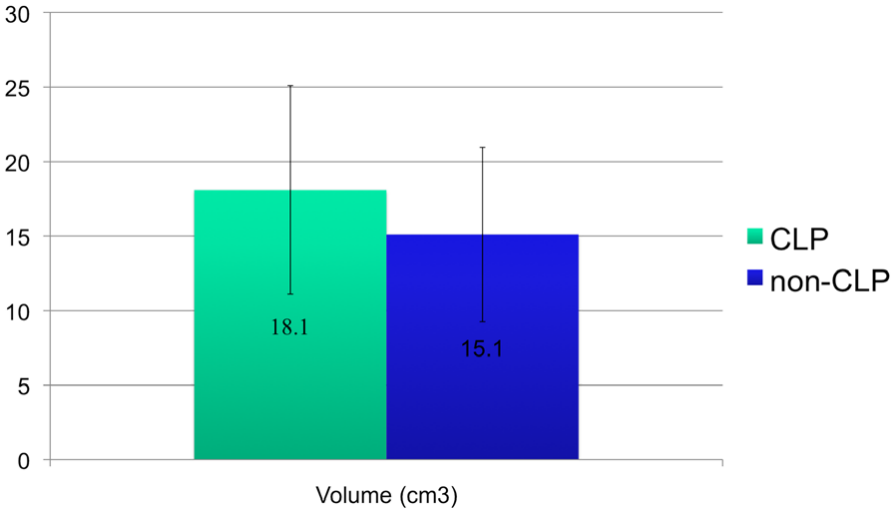
Graph depicting airway volume in CLP vs. non-CLP.

The airway volumes were not significantly different between the cleft and non-cleft groups (P = .07). The cleft group showed an average volume of 18.1 cm^3^ with a standard deviation of 7 cm^3^ and the non-cleft group showed an average volume of 15.1 cm^3^ with a standard deviation of 5.8 cm^3^ (Fig. 2).

**Figure 3:**
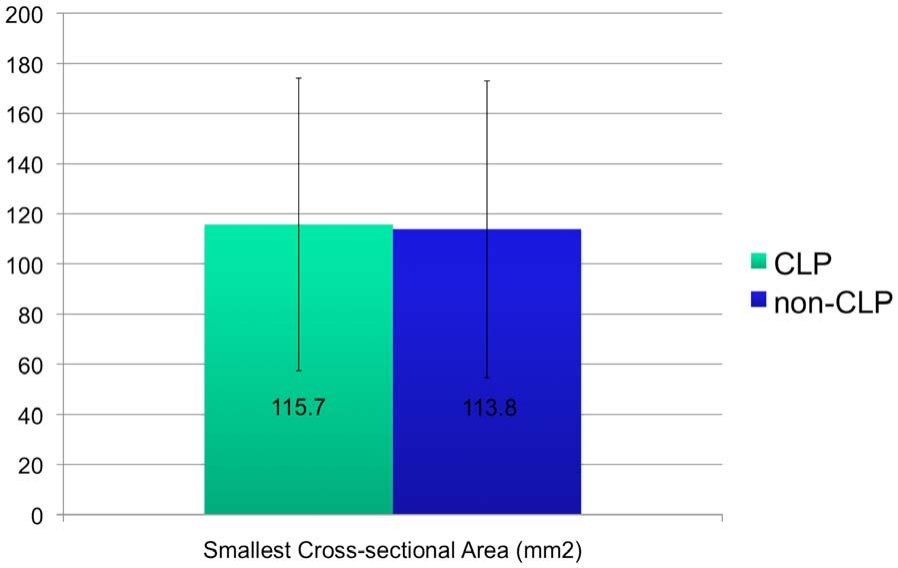
Graph depicting smallest cross-sectional area not significantly different in CLP vs. non-CLP.

The smallest cross sectional areas were not significantly different between the cleft and non-cleft groups (P = .45). The cleft group showed an average cross sectional area of 115.7 mm^2^ with a standard deviation of 58.5 mm^2^ and the non-cleft group showed an average of 113.8 mm^2^ with a standard deviation of 59.2 mm^2^ (Fig. 3). The location of the smallest cross sectional area for the cleft group was the oropharynx in 17 out of 19 of the cleft cases and 18 of the 19 non-cleft cases while the remaining in both groups was at the junction of the oropharynx and hypopharynx (Fig. 1).

**Figure 4:**
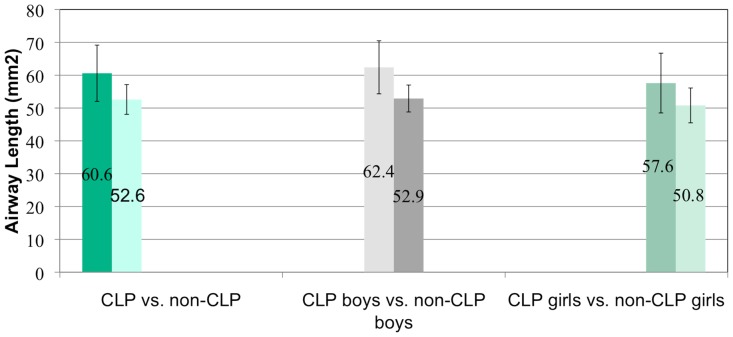
Comparisons of airway length in CLP vs. non-CLP, CLP boys vs. non-CLP boys and CLP girls vs. non-CLP girls.

The airway length was significantly different between the cleft and non-cleft groups (P = .004). The cleft group had an average length of 60.6 mm with a standard deviation of 8.6 mm and the non-cleft group had an average length of 52.6 mm with a standard deviation of 4.5 mm (Fig. 4 and Table 1). More specifically, the cleft and non cleft groups were assessed based on age. The males with clefts had an average airway length of 62.4 mm with a standard deviation of 8.1 mm and non-cleft males had an average length of 52.9 mm with a standard deviation of 4.1 mm (P = .01). The females with clefts had an average airway length of 57.6 mm with a standard deviation of 9.1 mm and the non-cleft females had an average airway length of 50.8 with a standard deviation of 5.3 mm (P = .20).

**Table 1:** Airway length measurements in CLP vs. controls.

	Average airway length (mm)	S.D.	p- value
CLP	60.6	8.6	
Controls	52.6	4.5	0.004
CLP boys	62.4	8.1	
Control boys	52.9	4.1	0.01
CLP girls	57.6	9.1	
Control girls	50.8	5.3	0.20

Average airway length in various groups.

**Figure 5:**
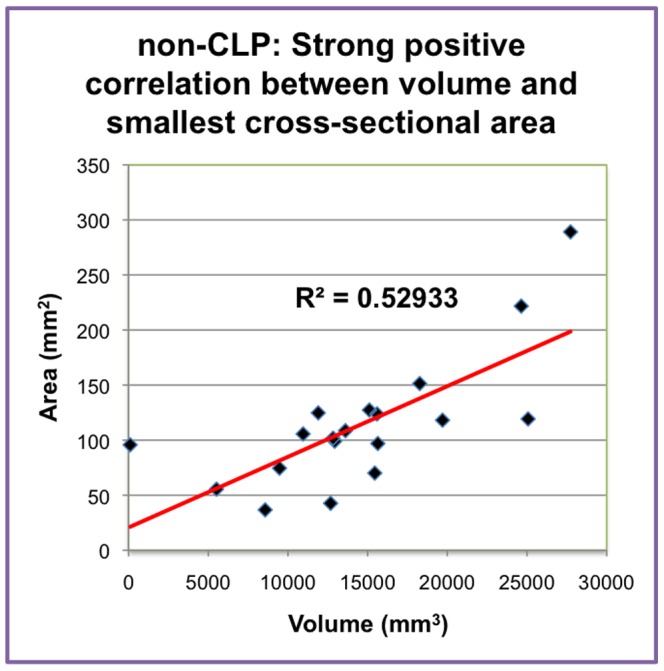
Graph depicting strong positive correlation between volume and smallest cross-sectional area in non-CLP.

A linear regression was used to model the relationship between volume and area for both CLP and non-CLP groups. A best-fit line was found on a scatter plot of airway volume and smallest cross sectional area. The r2 value was 0.37 and 0.53 (Fig. 5) for CLP and non-CLP, respectively. The CLP group demonstrated a weaker (less linear) relation between volume and cross-sectional area. In CLP individuals, a greater airway volume did not necessarily correlate to a proportionately larger cross-sectional area. Therefore, it was possible to use a ratio of the cross-sectional area to volume in order to compare the CLP group with the non-CLP group. The average ratio of cross-sectional area to volume was 0.0064 for the CLP group and 0.0076 for the non-CLP group. A one-tailed T test determined that there were no significant differences in the smallest cross-sectional area to volume ratio.

## Discussion

The goal of the study was to develop a reliable 3-D analysis to measure certain characteristics of the pharyngeal airway in children with CLP and compare the findings to a non-CLP control group, matched for age and sex. To the best of our knowledge, this is the first 3-D study to measure airway volume and smallest cross sectional area in children with CLP. Previous CBCT studies have assessed pharyngeal airway volume in healthy children with retrognathic mandible versus normal growth pattern, the oropharyngeal airway in children with class III malocclusion, and age related changes in the airway in children versus adults [28], [29], [30]. In our study, we hypothesized that children with CLP had smaller pharyngeal airways compared to non-CLP control group. Our data demonstrated that there was no significant difference in pharyngeal airway volume and smallest cross sectional area but that the airway length was significantly longer in CLP children when compared to non-CLP children.

Kim et al [30] measured the total airway volume for children (age range = 10.50–12.92 years) to be 20.96 cm^3^ with a standard deviation of 3.6 cm^3^. In our study, the mean airway volume was 18.1 cm^3^ (SD = 7.0 cm^3^) and 15.1 cm^3^ (SD = 5.8 cm^3^) for the cleft palate and non-cleft palate groups, respectively.

The mean smallest cross-sectional area for CLP individuals was 115.7 mm^2^ (SD = 58.4 mm^2^) and for controls 113.8 mm^2^ (SD = 59.2 mm^2^). The large standard deviations signify a wide range of values for cross-sectional area. This may be attributed to the age range of patients and the varying levels of growth for each patient. Abramson et al [28] found that the average smallest cross-sectional area in children age 6–11 was 82.9 mm^2^ (SD = 16.5 mm^2^) and for adolescents 12–16 was 122.2 mm^2^ (SD = 39.3 mm^2^). They did not find any significant differences in airway volume or minimum cross-sectional area between boys and girls. They also found that there was a significantly longer airway length in the study group compared to the control group but there was no difference in airway length between boys and girls. Ronen et al [31] found a differential growth in airway length in boys and girls during puberty resulting in significantly longer airway in boys (ages 14–19). In our study, there was a significantly longer airway in the CLP group compared to non-CLP group and in CLP boys compared to non-CLP boys. There were no significant differences in airway length between boys and girls in the age group studied.

Our study had limitations that may have contributed to the rejection of our hypothesis. This was a retrospective study with sample size limited to a small number of previously obtained scans of children with CLP. The respiratory cycle was not controlled while the scans were obtained. Respiration is a dynamic action that may not be accurately depicted in a static 3-D image. The images were taken after maxillary expansion, and it is possible that the larger airway volume found in CLP children could be the result of the expansion. Future studies should compare the volume in different regions of the pharynx (naso-, oro- and hypopharynx) to elucidate precisely where the airway is larger and should also explore airway length differences between CLP and non-CLP.
